# Clinicopathological and Immunological Changes in Indian Post Kala-Azar Dermal Leishmaniasis (PKDL) Cases in relation to Treatment: A Retrospective Study

**DOI:** 10.1155/2015/745062

**Published:** 2015-05-18

**Authors:** Neena Verma, Sanjiv Bimal, Vidya Nand Rabi Das, Krishna Pandey, Dharmendra Singh, Chandra Shekhar Lal, Ashish Kumar Singh, Prabhat Kumar Sinha, Pradeep Das

**Affiliations:** Rajendra Memorial Research Institute of Medical Sciences, Indian Council of Medial Research (ICMR), Agamkuan, Patna 800 007, India

## Abstract

Post-kala-azar dermal leishmaniasis (PKDL) is an important factor in kala-azar transmission; hence its early detection and assessment of effective treatment is very important for disease control. In present study on 60 PKDL cases presented with macular, mixed papulonodular, or erythematous lesions, *Leishmania parasites* were demonstrated microscopically in 91% of papulonodular and 40% of macular lesions. Cellular infiltrates in skin biopsy imprint smears from lesions were mononuclear cells, 25–300/OIF (oil immersion field), predominantly histiocytes with vacuolation, many lymphocytes, some plasma cells, and *Leishmania* amastigotes 0–20/OIF. Cases with no demonstrable parasites were diagnosed on the basis of past history of VL, lesion's distribution, cytopathological changes, and positive DAT (86.83%). Following antileishmanial treatment with SAG, papulonodular forms of PKDL lesions disappeared clinically but microscopically the mononuclear cells (20–200/OIF) persisted in the dermal lesions. Response observed in macular PKDL lesions was poor which persisted both clinically and cytopathologically. Follow-up of PKDL will assess the effectivity of treatment as either disappearance of lesions or any relapse. Studies on involvement of immunological factors, that is, certain cytokines (IL-10, TGF-*β*, etc.) and chemokines (macrophage inflammatory protein, MIP 1-*α*, etc.) in PKDL, may provide insight for any role in the treatment response.

## 1. Introduction

Leishmania, a protozoan parasite, is the causative agent of various forms of leishmaniasis, like visceral form (VL), mucocutaneous form (MCL), and cutaneous form (CL), of which VL is fatal. Postkala-azar leishmaniasis (PKDL) is a cutaneous form of leishmaniasis and usually occurs one to several years after apparent cure of VL caused by* Leishmania donovani *[1]. In some cases, previous history of symptomatic VL may be absent [2] indicating the possibility of subclinical infection. PKDL is common in India where it occurs in 6–20% of kala-azar (VL) cases following its attack [3]. It is characterized by hypopigmented macules (discrete or confluent) all over body sparing palm, soles, scalp, and axillae and erythematous papules or nodules predominantly over face.

PKDL has long been suspected as a potential reservoir of kala-azar infection. It is considered an important factor in disease transmission between kala-azar outbreaks in India [4]. Since PKDL cases harbour leishmania parasites superficially in the skin lesions, it is easy for the vector sandfly to pick up the parasites from the lesions and therefore it is considered an important source for transmission of the disease. Hence, for effective control of VL and to interrupt the transmission of kala-azar infection, its reservoir host PKDL needs to be detected early and treated adequately. The present study was undertaken in PKDL cases from VL endemic areas of Bihar, India, with objective to detect the different forms of PKDL cases and to correlate the cytopathological changes and parasite load in their dermal lesions with immunological changes and clinical response after full course of treatment with sodium antimony gluconate (SAG).

## 2. Materials & Methods

PKDL cases (*n* = 60) from endemic zones of Bihar were included in this study for parasite load, pathological changes in the skin, and immune response before and after treatment with sodium antimony gluconate (SAG). All cases were examined for any past history of kala-azar and details of its treatment, pattern of distribution, type, duration, site, size, coalescence, and sensation of skin lesions present. The study was approved by Institutional Ethical Committee and written informed consent was obtained from all the subjects.

Skin biopsies were collected aseptically from edges of the dermal lesions. One part was put in the biphasic culture media and from other part, biopsy imprint smears were prepared on clean glass slides and stained with Leishman/Giemsa stain. The remaining part was fixed in 10% buffered formalin for histological sections.

Venous blood was collected for haematological (TC, DC of WBC, Hb%, and total platelet counts) and the immunological tests, that is, direct agglutination test (DAT, [5]) and migration inhibition factor (MIF, [6]).

### 2.1. Skin Biopsy/Smear Collection from PKDL Patients and Microscopy

Skin biopsies/smears were collected from the dermal lesions of PKDL patient aseptically who had given his/her consent before screening for the study.

Two clean glass slides were labelled by diamond pencil with laboratory number, which was also noted on patient's request form for skin biopsy/smear filled up and sent by the clinician to the Pathology laboratory. Selected site of skin lesion was sterilized with 70% alcohol swab and allowed to dry. Effort was taken to blanch the area by pressing with forceps on the base of the site. Using sterile scalpel with surgical blade and smooth end forceps, a tiny piece of skin tissue was cut superficially to avoid scarring. Imprint smears of skin tissue were prepared on the grease-free glass slides labelled with diamond pencil. After 5 minutes of soft pressure, the cut area of skin was covered with medicated dressing (handyplast) which is ventilated and containing antiseptic 0.2% Nitrofurazone.

For collection of skin smear, the skin is grasped between the thumb and forefinger of the hand until the site is blanched. Approximately 5 mm long and 1-2 mm deep incision was made with scalpel blade. The site of the incision is scraped inside with scalpel to obtain tissue fluid and pulp. Smears are made on the grease-free clean glass slides and prenumbered with diamond pencil.

After collection, skin smear slides were immediately fixed with methyl alcohol and allowed to dry at room temperature. Slides were stained with diluted (1 : 9) Giemsa stain for 25 minutes. Smears are examined under the oil immersion objective of the microscope for demonstration of* Leishmania *parasites [7]. Gradation of the parasite load in skin smear was done as per the WHO criteria [8].

### 2.2. Preparation of DAT Antigen [9]

Antigen prepared from crude* Leishmania donovani* promastigotes (96 h stationary-phase cultured promastigotes of a reference strain of* L. donovani *(WHOM/IN80/DD8), in a monophasic medium with 20% foetal calf serum) was used in the DAT study to observe the antibody titre [10]. Following trypsin treatment, the harvested promastigotes were washed twice with Locke's solution (154 mM NaCl, 2 mM CaCl_2_, and 2 mM NaHCO_3_, with 0.25% glucose) and then fixed, for 18–20 h, in 2% (w/v) formaldehyde in Locke's solution. After washing in citrate saline (0.5 M NaCl and 0.05 M sodium citrate at pH 7.4), the parasites were stained with 0.02% Coomassie Brilliant Blue, washed again with citrate saline, resuspended, at a concentration of 7.5 × 10^7^/mL, in citrate saline containing 0.43% (w/v) formaldehyde, and then stored at 4°C until used.

### 2.3. Optimizing and Evaluating the Performance of the DAT

To determine a suitable threshold titre for seropositivity in the DAT and then to explore the sensitivity and specificity of the test, samples of the fingerprick blood were collected, onto 1-mm-thick filter paper. These subjects were parasitologically confirmed, active and untreated cases of VL (*N* = 108), the cases of tuberculosis (*N* = 10), malaria (*N* = 10), leprosy (*N* = 10), and filariasis (*N* = 10), and apparently healthy controls who lived either in the nonendemic districts (*N* = 452) or, adjacent to an endemic area, in Muzaffarpur district (*N* = 189). Bone marrow and/or splenic aspirates from each of the VL cases were investigated. Amasigotes had been found in the aspirates, and when the blood samples had been collected, each of these cases had splenomegaly or recent history of fever.

The blood spots were allowed to dry before 0.5 cm-diameter circles (each covered in dried blood) were cut from the dried filter paper sheets. Each of the circles was then immersed in 165 *µ*L saline (0.9% NaCl, pH 7.4) to give a solution of serum diluted 1 : 50 in saline [11].

The dilution sera were then investigated in DAT [11], which were performed in microtitre plate (Nunc, Roskilde, Denmark) that each had 96, V-shaped wells. In these tests, saline supplemented with 0.2% (w/v) gelatin and 0.78% (v/v) *β*-mercaptoethanol was dispensed into the wells of the plate (at 50 *µ*L/well), so that a 50 *µ*L sample of each test serum (already diluted 1 : 50 during its elution from a filter-paper circle of dried blood) could be further diluted, in twofold series. Some wells (the first in each row) were left serum-free, as controls. Reference sera, known to be positive or negative for antileishmanial antibodies, were also included, as controls. The suspension of stained parasites was then dispensed into the wells, at 50 *µ*L/well, before the plates were covered and the contents of each well were mixed (by gentle swirling each plate across the bench top for 30 s). After incubation at 18–22°C for 18 h, each plate was read by eye, the end-point titre for each test serum being the highest dilution giving visible agglutination (i.e., blue mats or blue dots, with or without fuzzy edges that were larger than those seen in the negative-control wells).

The sensitivity of the DAT was evaluated by comparing the results of the VL/PKDL cases with those of the apparently healthy controls. The specificity of the test was evaluated, however, by comparing the results of the VL cases with those of the patients who had nonleishmanial infections. The way in which the titre used as the threshold for positivity influenced the sensitivity and specificity of the DAT and the relationship between sensitivity (true positivity) and specificity (false negativity) were used to identify the optimal threshold titre. The DAT was then used, with this optimal titre, to see how useful it would be in detecting subclinical infections.

Migration inhibition factor (MIF) assay was evaluated in PKDL and active kala-azar (as control) cases to measure the cell mediated immunity (CMI) status [12]. In brief, lymphocytes were separated from heparinized blood and cells were washed three times with RPMI-1640. Finally, the aliquots were distributed in 4 cells cut in a preparation of agarose in a petri dish (15 × 90 mm). Two wells were filled with soluble or LD antigen while the rest were filled with the medium (control wells). The petri dish was incubated overnight at 37°C in humidified chamber with 5% CO_2_. Next day, the cells that had migrated under the agarose were fixed and stained. The diameter of the migration areas was measured to calculate the MI [13].

All these tests were done before and after treatment with SAG (10 mg/kg/day for 90 days).

Biopsy imprint smears were examined under microscope (Oil immersion and high power fields) for detection of leishmania parasites and cytological changes in skin lesions of postkala-azar dermal leishmaniasis during different phases of collections.

## 3. Results

Out of 60 PKDL cases, 39 were males and 21 females aged between 7 and 57 years. Only 54 cases had past history of VL of duration ranging between 3 months and 10 years, and the remaining 6 cases had no past history of VL. The duration of dermal lesions in these cases was in range of 2 months to 20 years. Twenty-five (41.67%) PKDL cases had only hypopigmented macular lesions, 15 (25%) cases had papuloerythematous lesion and 20 (33.33%) cases had mixed lesions (with all macular, erythematous, and papulonodular type) (Table 1).

Microscopically leishmania parasites were detected in 95% of mixed papulonodular lesions whereas only in 40% of cases with macular lesions. PKDL cases, where no parasites were demonstrated microscopically, were diagnosed on basis of clinical presentation, past history of kala-azar and its treatment, histopathological changes and cytological findings in the imprint smears of biopsy from skin lesions, and DAT results. The leishmania parasite density in the positive cases was 0–20/OIF of the biopsy imprint smears. They were found mostly (88%) extracellular. Most of the parasites (73%) were of proper morphology with healthy looking but few (27%) were with faint cytoplasmic border.

### 3.1. Cytopathological Changes

The dermal infiltrates observed in the biopsy imprint smears consisted of mononuclear cells (25–300/OIF), a mixture of histiocytes, lymphocytes, and occasional plasma cells. In papulonodular lesions, the histiocytes were predominant cells with many activated macrophages having vacuolated appearance and scattered epitheloid cells and plasma cells. In hypopigmented macular lesions, the predominant cells were lymphocytes with some histiocytes and scarce plasma cells. DAT was positive in 86.83% of PKDL cases with titre ranging between 1 : 800 and 1 : 25600 and MIF was positive (>20%) in 70% of PKDL cases studied, with result range of 17–38% (mean 27.9%). All the ten active kala-azar cases were negative for migration inhibition factor (<20%) (Table 2).

Pathologically, after schedule treatment with SAG, papulonodular form of all PKDL cases was negative for leishmania parasites except one. Mononuclear cells in imprint smears came down to 20–200 cells/OIF with many histiocytes, increase of lymphocytes, and occasional plasma cells. Although the lesions improved clinically, the cytological changes in the dermal lesions persisted even after treatment. In case of macular lesions, there were no significant changes in the lesions both clinically and pathologically after treatment. The number of mononuclear cells was in the same range (15–50/OIF). Thus, it was very difficult to decide the complete cure in these forms. After treatment, the DAT titre was in the range of 1 : 800 to 1 : 6400 whereas MIF was positive in all the ten PKDL cases with value of range 33–65% (mean 46.8%). In 3 PKDL cases, the lesions persisted cytopathologically even after one year of treatment. In all PKDL cases, no significant haematological changes were observed in either group except increase of eosinophils (ranged 10–30%) of total leucocytes.

## 4. Discussion

Persistence of Kala-azar in endemic form in Bihar and other affected areas and the absence of zoonotic reservoir of kala-azar infection as observed in several studies [14, 15] raised a possibility of existence of human reservoir in the form of PKDL or asymptomatic carriers in the community, as they harbour leishmania parasite in the skin or blood. They may help in persistence of transmission of infection and spread of the disease.

In our study, diagnosis of PKDL was confirmed pathologically by microscopic demonstration of parasites in the skin lesions of patients presented with clinical features of PKDL. Clinical diagnosis was based on characteristic skin lesions in the patients treated for kala-azar. Diagnosis may be problematic in cases with long clinical interval with up to 30 years, being reported between completed treatment of Kala-azar and the appearance of skin lesions [16–18].

A definitive diagnosis is demonstration of parasites in the skin lesions, but parasites are often scanty and require a prolong research. In our study, thin skin biopsies were collected superficially from suspected PKDL lesions to prepare imprint smears for microscopic demonstration of leishmania parasites resulting in high positivity of 95% in mixed papulonodular lesions (Table 1). This shows that leishmania parasites are most abundant just beneath the epidermis and it should be taken into consideration when taking biopsy and making smears [2]. Culture is often difficult due to risk of bacterial and fungal contamination in the skin tissues [19]. Taking biopsy and smears is often resented in young children since face is the most common site of the lesion. Other techniques such as PCR are promising but yet to be assessed for their application in PKDL diagnosis since limited studies have been conducted in these cases [19, 20]. DAT is useful in differential diagnosis, for example, with leprosy [21], although a negative result does not exclude kala-azar or PKDL, as found in our present study also.

Studies of the immune responses showed that unlike kala-azar patients, in most (70%) of the PKDL cases, T-cells reacted in response to leishmania antigen as observed in MIF assay and there was no suppression of CMI response in them. In contrast to patients with kala-azar who show no response to leishmania antigen, patients with PKDL develop some degree of immunological competence to the parasite, but the response is insufficient to bring about its elimination from the skin [22].

In the present study, we have observed that, after scheduled course of treatment with SAG in PKDL cases, although skin lesions disappeared clinically in papulonodular and erythematous cases with no parasite in most of the cases, the dermal pathology with cellular infiltration persisted as observed microscopically in the imprint smear cytology of skin biopsy collected from the same site. But the density and number of the mononuclear cells per oil immersion field (OIF) of the microscope were comparatively less (20–200/OIF) than before initiation of the treatment (25–300/OIF) (Table 2). This indicates that the cases might have responded clinically, but the cytological response within the dermal lesion is gradual. Hence, microscopic examination for imprint smear cytology along with leishmania parasite detection in thin skin biopsy from PKDL lesion site should be followed at the interval of 3–6 months to ensure the complete treatment both clinically and pathologically. In macular cases, no conspicuous difference was observed in the lesions both clinically and microscopically in the imprint smear cytology and in DAT results. Hence, it is very difficult to decide the complete cure in these forms of PKDL.

Further studies on the involvement of certain immunological factors such as some cytokines (IL-10, TGF-*β*, IFN-*γ*, etc.) and chemokines (macrophage inflammatory protein, MIP 1-*α*, Rantes, etc.) in the dermal lesions of PKDL cases (before and after chemotherapy) may provide insight for any role in the disappearance of lesion and treatment response.

Some of the PKDL cases required prolonged treatment and these cases are being followed up for two years, cytopathologically with parasite detection and correlating with clinical and immunological changes to observe any redevelopment of dermal lesions or complete recovery. There is no satisfactory test of cure in macular PKDL cases as parasites are often difficult to find in smears or biopsies from these skin lesions and there are no conclusive immunological findings in relation to treatment in these cases that ensure the response of the specific chemotherapy. The PCR may prove an important tool in this respect but is yet to be evaluated [23].

## Figures and Tables

**Table 1:** Showing age, sex, duration of past history of Kala-azar and leishmania positivity in various types of PKDL cases.

Type of lesion	Hypopigmented macular	Papuloerythematous	Mixed macular Papulonodular lesion	Total
Number of PKDL Cases	25	15	20	60
With past history of Kala-azar	21	14	19	54
No past history of Kala-azar	04	01	01	06
Mean age range (years)	14.15 (7–30)	21.78 (10–36)	26.86 (17–57)	20.45 (7–57)
Sex (male/female)	15/8	6/9	18/4	39/21
Duration of past History of Kala-azar in mean (range)	5 years (4 months–10 years)	8.95 years (1–22 years)	8.75 years (3–18 years)	7.5 years (4 months–22 years)
Leishmania parasite positivity number (%)	10 (40%)	13 (86.67%)	19 (95%)	40 (66.67%)
DAT positivity (%) cases titre	83.4% (1 : 800–1 : 6400)	83.33% (1 : 1600–1 : 6400)	93.75% (1 : 12800)	86.83% (1 : 800–1 : 12800)

**Table 2:** Clinicopathological changes in PKDL lesions in relation to treatment and immune response.

Parameters	Before treatment	After treatment
Clinical changes	Nodulo-papular	Absent or low papular lesion
	Erythematous over whole face	Over chin only
	Macular over all body	Present but slightly fainter

Microscopic findings		

Leishmania parasite Positivity with density	Positive in 91% of papulonodular cases and 40% of macular cases. Mostly extracellular, only few intracellular. (0–20/OIF)	Negative in all except one.

Cytological changes	Mononuclear cells (+++)	Number decreased (++)
	25–300/OIF, mostly clustered	20–200/OIF, Scattered.
	Histiocytes predominant (15–250/OIF)	Many Histiocytes (10–140/OIF)
	Lymphocytes: 10–40/OIF	Lymphocytes: 10–60/OIF
	Plasma cells, epitheloid cells occasional	Plasma cells scarce
	Many activated macrophages with vacuolated appearance	Some activated macrophages with vacuolation present

Immunological response	DAT—75% positive	DAT—66% positive
	MIF +ve 70%, value 17–38%	MIF +ve all, value 33–65%

## References

[1] Sen Gupta P. C., Banerjee J. C., Bhattacharya P. B. (1960). *Handbook of Tropical Diseases*.

[2] El Hassan A. M., Ghalib H. W., Zijlstra E. E. (1992). Post kala-azar dermal leishmaniasis in the Sudan: clinical features, pathology and treatment. *Transactions of the Royal Society of Tropical Medicine & Hygiene*.

[3] Bryceson A. D. M. (1996). Leishmaniasis. *Manson's Tropical Diseases*.

[4] Thakur C. P., Kumar K. (1992). Post kala-azar dermal leishmaniasis: a neglected aspect of kala-azar control programmes. *Annals of Tropical Medicine and Parasitology*.

[5] Harith A. E., Kolk A. H. J., Kager P. A. (1986). A simple and economical direct agglutination test for serodiagnosis and sero-epidemiological studies of visceral leishmaniasis. *Transactions of the Royal Society of Tropical Medicine & Hygiene*.

[6] Bloom B., David J. (1973). *In Vitro Methods in Cell Mediated and Tumor Immunity*.

[7] Verma N., Singh D., Pandey K. (2013). Comparative evaluation of PCR and imprint smear microscopy analyses of skin biopsy specimens in diagnosis of macular, Papular, and mixed Papulo-nodular lesions of post-Kala-Azar dermal Leishmaniasis. *Journal of Clinical Microbiology*.

[8] Chulay J. D., Bryceson A. D. (1983). Quantitation of amastigotes of *Leishmania* donovani in smears of splenic aspirates from patients with visceral leishmaniasis. *American Journal of Tropical Medicine and Hygiene*.

[9] Bimal S., Das V. N. R., Sinha P. K. (2005). Usefulness of the direct agglutination test in the early detection of subclinical *Leishmania* donovani infection: a community-based study. *Annals of Tropical Medicine & Parasitology*.

[10] Harith A. E., Kolk A. H. J., Leeuwenberg J. (1988). Application of direct agglutination test for field studies of visceral leishmaniasis. *Journal of Clinical Microbiology*.

[11] El Harith A., Slappendel R. J., Reiter I. (1989). Application of a direct agglutination test for detection of specific anti-Leishmania antibodies in the canine reservoir. *Journal of Clinical Microbiology*.

[12] Hamlin A. S. (1981). *Techniques in Clinical Immunology*.

[13] Das V. N. R., Ranjan A., Bimal S. (2005). Magnitude of unresponsiveness to sodium stibogluconate in the treatment of visceral leishmaniasis in Bihar. *National Medical Journal of India*.

[14] Zahar A. R. (1979). *Studies on Leishmaniasis Vector/Reservoirs and Their Cntrol in the Old World*.

[15] Bhattacharya A., Ghosh T. N. (1983). A search for *Leishmama* in vertebrates from kalaazar-affected areas of Bihar, India. *Transactions of the Royal Society of Tropical Medicine & Hygiene*.

[16] Thakur C. P. (1984). Epidemiological, clinical and therapeutic features of Bihar kala-azar (including post kala-azar dermal leishmaniasis). *Transactions of the Royal Society of Tropical Medicine and Hygiene*.

[17] Rashid J. R., Chunge C. N., Oster C. N., Wasunna K. M., Muigai R., Gachihi G. S. (1986). Post-kala-azar dermal leishmaniasis occurring long after cure of visceral leishmaniasis in Kenya. *East African Medical Journal*.

[18] Muigai R., Gachihi G. S., Oster C. N. (1991). Post kala-azar dermal leishmaniasis: the Kenyan experience. *East African Medical Journal*.

[19] Osman O. F., Oskam L., Kroon N. C. M. (1998). Use of PCR for diagnosis of post-kala-azar dermal leishmaniasis. *Journal of Clinical Microbiology*.

[20] Merdith S. E. O., Zijlstra E. E., Schoone G. J. (1993). Development and application of the polymerase chain reaction for the detection and identification of Leishmania parasites in clinical material. *Archives de l'Institut Pasteur de Tunis*.

[21] El Hassan A. M., Hashim F. A., Abdullah M., Zijlstra E. E., Ghalib H. W. (1993). Distinguishing post-kala-azar dermal leishmaniasis from leprosy: experience in the Sudan. *Leprosy Review*.

[22] Zijlstra E. E., El-Hassan A. M., Ismael A. (1995). Endemic kala-azar in Eastern Sudan: post-kala-azar dermal leishmaniasis. *The American Journal of Tropical Medicine and Hygiene*.

[23] Zijlstra E. E., El-Hassan A. M. (2001). Leishmaniasis in Sudan. 4. Post kala-azar dermal leishmaniasis. *Transactions of the Royal Society of Tropical Medicine and Hygiene*.

